# Dementia and Lifestyle Risk Factors in Sub‐Saharan Africa: Exploring Epidemiological Insights

**DOI:** 10.1002/alz.090056

**Published:** 2025-01-09

**Authors:** Tobi Nifemi Olajide, Isaac Babawale, Ifeoluwa Oluwasegun Oduguwa, Rachel Dada, Evelyn Ogungbemi, Napoleon Tejiri, Adeosun Ololade, Chukwuebuka Stanley Asogwa, Adamma Njoku, Oghenemaro Jerry‐Ogeme, Joshua Alabi, Teslim Adepoju, James Iziren, Deborah Ogundijo, Oluwatimilehin Oladapo, Habib Shobanke, Chibuike Eziechina, Adesola Ogunniyi

**Affiliations:** ^1^ College Research and Innovation Hub, Ibadan Nigeria; ^2^ College of Medicine, University of Ibadan, Ibadan Nigeria; ^3^ Rostov State Medical University, Rostov‐on‐Don, Southern Russia Russian Federation; ^4^ College of Medicine, University of Ibadan, Ibadan, Oyo Nigeria

## Abstract

**Background:**

The emergence of dementia as a global health challenge necessitates an exploration of its unique epidemiological patterns and risk factors in Sub‐Saharan Africa (SSA). Amid a growing elderly population, SSA presents an intriguing paradox of lower‐than‐expected dementia prevalence, prompting a comprehensive review of epidemiological nuances, lifestyle risk factors, cultural influences, and protective factors. This study critically assessed the current state of dementia research in SSA, aiming to inform tailored interventions and policies.

**Method:**

A comprehensive search was initiated across electronic databases, including PubMed, MEDLINE, PsycINFO, and Web of Science, utilizing keywords such as “Dementia,” “lifestyle factors,” “epidemiology,” “risk factors,” and “Sub‐Saharan Africa.”

To ensure relevance and currency, only peer‐reviewed studies published from 2013 till 2024 were considered. The screening process commenced with a thorough evaluation of titles and abstracts using the Rayyan software. 69 papers met the inclusion criteria upon full text review. Data extraction was executed to compile pertinent information from the selected studies.

**Result:**

Dementia prevalence in Sub‐Saharan Africa (SSA) is diverse, influenced by factors ranging from dietary habits to socio‐economic status, with rates ranging from 2.3% to 20% in community‐based studies. Lifestyle factors, including diets rich in antioxidants and cultural practices, contribute to the complex tapestry of dementia epidemiology. Physical activity’s protective role in cognitive decline was underscored by a meta‐analysis, but regional variations necessitate tailored interventions. Cognitive engagement, influenced by education and social participation, emerged as a potential protective factor. Cultural practices, communal living, and environmental factors shape the dementia landscape, with communal responsibilities and traditional diets showing protective effects. Spousal relationships and psychosocial support also demonstrate protective effects, emphasizing the need for addressing healthcare infrastructure gaps and cultural adaptations in effective dementia risk reduction strategies.

**Conclusion:**

Despite a growing elderly population, Sub‐Saharan Africa currently experiences surprisingly low dementia rates. This study suggests prioritizing childhood education, community‐based initiatives for cognitive and physical activity, and implementing policies to reduce dementia risk. Strengthening healthcare infrastructure, investing in research, and developing culturally sensitive interventions are crucial for addressing the impending dementia crisis. Bridging knowledge gaps and implementing comprehensive strategies can pave the way for a dementia‐resistant future in the region.

